# Nocebo Effects of Clinical Communication and Placebo Effects of Positive Suggestions on Respiratory Muscle Strength

**DOI:** 10.3389/fpsyg.2022.825839

**Published:** 2022-03-11

**Authors:** Nina Zech, Leoni Scharl, Milena Seemann, Michael Pfeifer, Ernil Hansen

**Affiliations:** ^1^Department of Anaesthesiology, University Hospital Regensburg, Regensburg, Germany; ^2^Department of Pediatrics, RoMed Klinikum Rosenheim, Rosenheim, Germany; ^3^Department of Anaesthesiology, Agaplesion Diakonieklinikum Hamburg, Hamburg, Germany; ^4^Department of Internal Medicine II (Cardiology and Pulmonology), University Hospital Regensburg, Regensburg, Germany

**Keywords:** nocebo effect, placebo effect, suggestion, respiratory muscle strength, spirometry, flow, pressure

## Abstract

**Introduction:**

The effects of specific suggestions are usually studied by measuring parameters that are directly addressed by these suggestions. We recently proposed the use of a uniform, unrelated, and objective measure like maximal muscle strength that allows comparison of suggestions to avoid nocebo effects and thus to improve communication. Since reduced breathing strength might impair respiration and increase the risk of post-operative pulmonary complications, the aim of the present study was to evaluate the effects of the suggestions on respiratory muscle power. Both the identification and neutralization of negative suggestions in the clinical context and stimulating suggestions could improve breathing force, a predictor of physical fitness and convalescence.

**Methods:**

In 50 healthy, adult volunteers, respiratory muscle strength was measured by maximal inspiratory and expiratory pressures, as well as by maximal inspiratory and expiratory flows. Baseline was compared to values after application of eleven suggestions, five out of clinical context, including memory of negative or positive past, risk information for informed consent, and a non-verbal suggestion. Six stimulating suggestions included self-affirmation, empowering words, a heroic mirror image, and an imagination.

**Results:**

All suggestions showed an impact on respiratory muscle strength, indicating placebo and nocebo effects. No single parameter could represent the breathing force in its complexity, however, trends and different specific aspects of it were measured. The strongest reaction was observed with the recall of a previous negative situation resulting in a reduction in expiratory flow to 96.1% of baseline (*p* = 0.041). After risk information, a decrease was observed in three of the parameters, with the highest extend in expiratory pressure by 4.4%. This nocebo effect was neutralized by adding positive aspects to the risk information. Every intended strengthening suggestion resulted in statistically significant increases of at least one parameter, with changes of up to 10% (e.g., MEP 110.3%, *p* = 0.001), indicating placebo effects. Here, expiration was more affected than inspiration. Sex was the only influencing factor reaching statistical significance, with stronger reactions in women.

**Conclusion:**

Respiratory muscle strength proved to be sensitive to suggestions with clinical context, as well as suggestions intended for stimulation. With this objective measurement, evaluation, and comparison of different suggestions is possible to help avoid nocebo effects. The demonstrated effect of supporting suggestions can be followed up and used in clinical practice.

## Introduction

The role of nocebo effects in medicine is increasingly recognized (Häuser et al., 2012; Colloca, 2017; Hansen and Zech, 2019). They can interfere with the well-being and health of the patients and can jeopardize the therapeutic efficacy. Nocebo effects and negative suggestions are often described as nonspecific. Nevertheless, they are usually tested in a very specific manner, namely according to the symptom addressed. For example, the word “pain” can induce or increase pain (Lang et al., 2005), the question about nausea can induce or aggravate nausea. Besides these specific consequences of negative communication, there are also more general effects, which are much more difficult to measure for their complexity (e.g., immunoreactions) or delayed effect (e.g., wound healing and recovery after an operation). We recently conducted two studies on the impact of suggestions from medical everyday life on maximal arm muscle strength, an objective measure from physiology research (Zech et al., 2019; Zech et al., 2020). This objective parameter allows the identification of the nature of a suggestion (positive or negative), the quantification of the effect, and their comparison, by applying a uniform outcome parameter for different suggestions. It enables the development, evaluation, and optimization of alternative formulations of the suggestion and thus ultimately communication improvement. In these studies, several non-verbal and verbal presentations, inter alia sentences that included the words pain and nausea, led to a significant reduction in maximal arm muscle strength interpreted as a general sign of a “weakening” of the patient. It is noteworthy that these effects on muscular performance were observed without specifically addressing muscle strength or physical activity and movement. However, any impairment in muscular performance has a high clinical impact. In times of fast-track surgery, patient unimpaired recovery is an interdisciplinary goal aimed at early and intensive mobilization, as well as physical and respiratory training. Muscular weakness must be prevented as it may cause complications such as stumbling and subsequent injuries. Additionally, impaired muscular strength could alter breathing and increase the risk of post-operative pulmonary complications such as atelectasis and pneumonia (Dronkers et al., 2008; Kulnik et al., 2014).

To clarify the validity of the latter apprehension of high clinical impact, we performed a study on effects of suggestions on breathing power, which will be reported here. Whereas dynamometry of arm abduction is a relatively clear measurement with defined and known muscles involved, breathing is a complex muscular function that includes movements of the diaphragm and thoracic muscles. Accordingly, several test parameters of pressure and flow, inspiration and expiration were evaluated. Some of the suggestions tested were identical to those analyzed for impact on maximal arm muscle strength. However, it is not only important to avoid nocebo effects and a weakening in patients by identifying the negative nature of a suggestion, by quantification of its effect, and by development of alternative, neutral formulations. In addition to greater awareness of negative influences and how to combat them, efforts can be made for a positive impact. Therefore, we also included and evaluated positive suggestions that could improve and increase breathing power and strengthen the patient. This approach could support communication for both prophylactic and therapeutic purposes. We hypotheses, that suggestion have impact on respiratory muscle strength.

## Materials and Methods

### Design and Participants

After approval of the local ethics committee (EC University of Regensburg, 13-101-0030), an experimental, prospective, cross-over, within participants study was carried out with 50 healthy volunteers after informed consent. The age of the participants was limited to 18–65 years. Exclusion criteria were language barriers or relevant health restrictions, particularly pulmonary disease (Scoring > II in the physical status classification of the American Society of Anaesthesiologists). Participation was without financial compensation. Enrolment of participants, data collection and evaluation were carried out by a medical student under supervision as part of a doctorial theses. Validity and reliability of the breathing parameters were achieved by extensive training and supervision of the performing student by the cooperating pulmonology lab.

### Measurement of Lung Function

Lung function can be tested and analyzed by various parameters. Spirometry that produces flow and volume values is used to diagnose and classify diseases such as asthma or chronic obstructive pulmonary disease (COPD), and to distinguish obstructive from restrictive lung problems. Measurement of respiratory muscle strength is used to assess risk and prevention of post-operative complications such as atelectasis or pneumonia. The most important parameters of the respiratory muscles are given by maximal flows and pressures.

In this study, the strength of the respiratory muscles was evaluated with two different non-invasive technics: spirometry and the measurement of airway pressure, according to the recommendations of the European Respiratory Society and the American Thoracic Society (American Thoracic Society/European Respiratory Society, 2002). Peak inspiratory and peak expiratory flow (PIF/PEF) in L/s, relevant for the description of muscle strength, were obtained by spirometry. The EasyOne-line™ spirometer (ndd Medizintechnik AG, Zürich, Switzerland) was used, measurements were recorded with Easyware software. This software gives immediate feedback about the quality of the breathing maneuver and initiates a repeat if needed. Furthermore, the two airway pressures “maximal inspiratory pressure” (MIP) representing the diaphragm, and “maximal expiratory pressure” (MEP) representing the abdominal, intercostal, and accessory musculature (Enright et al., 1994) were measured in cmH_2_O. For these tests, the device PTS2000 Version 4.0 (Puritan Bennett, Pleasanton, CA, United States) and the software BreathLab PTS were used. An accuracy of 2.5% is reported for spirometry and of 1% for pressure measurements (Graham et al., 2019; Aymerich et al., 2021). The tests took place after information, demonstration, and practicing of the maneuver in a quiet, designated room at the university hospital. To guarantee high standardization, participants listened to recorded instructions for the prescribed breathing maneuver. The measurements of airway pressures were accompanied by “Fully exhale. Put the device in your mouth and now inhale to the maximum. Continue breathing normally. Take a deep breath. Now put the device in your mouth again and exhale to the maximum. Continue breathing normally.” Spirometry was guided by “Take a deep breath. Put the device in your mouth and exhale to the maximum now. Keep going. And now inhale to the maximum.”

### Tested Suggestions and Application

The examiner gave verbal suggestions face to face, and visual suggestions were shown on a tablet. Five suggestions were taken from everyday clinical life during a hospital stay. Six further suggestions were tested, all of which had an expected positive influence on pulmonary muscle strength.

After the evaluation of a short medical history to exclude relevant pre-existing disease, the breathing maneuver was demonstrated, explained, and practiced with the test person. After hearing the prescribed and recorded instructions, the participants performed baseline values for spirometry and pulmonary pressure, each three times. Three baseline measurements were made at the beginning of the test and four others in pairs of two during the test to recognize learning effects or exhaustion. During the procedure, intended breaks were kept and additionally given, whenever the test person reported being out of breath, hyperventilation, or dizziness, as well as if baseline values deviated by more than 10%. The suggestions listed in Table 1 were presented personally by the examiner, followed by recorded instruction. To help the participant keep the suggestion in mind, the examiner repeated a short version during the breathing maneuver. Suggestions were given in a randomized order, using the Research Randomizer Version 4.0 (Urbaniak and Plous, 2013). The only exception was the repetition of the own empowering word, and this suggestion was tested at last. To avoid influencing or accumulating effects, each suggestion was separated from the next one through simple arithmetic tasks. The whole test took about 60 min, within a tolerable framework.

**TABLE 1:** Wording of verbal suggestions and description of non-verbal suggestions taken from clinical practice and to improve breathing power.

Situation in the past	Version A	“Remember a situation, where something went really wrong. Everyone was disappointed in you, including yourself. This was terrible. You were really ashamed.”
	Version B	“Remember a situation, when you were really successful and entirely satisfied with yourself. Everything went so well—totally perfect.”
Risk information for informed consent	Version A	“If you wish, we can place a pain catheter, with the risk of infection, allergic reaction, and damage to blood vessels or nerves.”
	Version B	“We have the option of a catheter to prevent discomfort. Even though there is a risk of infection, allergic reaction, or damage to blood vessels or nerves you will have to take fewer pills, are more mobile, feel and recover better, and maybe can go home sooner.”
Transportation to the OR		“Imagine you are a patient in hospital. You are lying in bed, being brought to the operation room. That is what you see.”
Self-affirmation (with circular massage of a point under the clavicula by two fingers)		“It has been found that performance and feelings have a lot to do with motion and body sensation. Please also do this movement. And now repeat the following sentence: Even if sometimes I am so stressed and tired that I run out of breath, I like myself and accept myself as I am.”
Empowering word	Given: fireball	“Perhaps there is a fitting word for all your energy and inner strength, the epitome of strength. Let me tell you a word like that: fireball!”
	Chosen by the participant	“Maybe you can find a much better word yourself. What would be such an empowering word for you? When you find something, just nod. Now say that word for yourself. You can also speak it out loud.”
	Repetition of the own empowering word	”You had previously found your own strong empowering word. Recall now and feel how it works in you. Think of your empowering word.”
Strengthening of self-perception	Picture of a cat, looking in a mirror and seeing a lion	“Please look at this picture. Now close your eyes and imagine that you look in a mirror. What would it be, what you see. Which animal, which hero would really give you strength? If you see your reflection in front of you, please nod briefly.”
Inflating a balloon		“Imagine, you have a balloon, you can inflate it. With every breath it gets bigger and bigger, until it is big enough for you to fly away with it. Take deep breaths and inflate firmly!”

*Four different parameters are measured to assess respiratory muscle strength after giving the suggestions face to face. To facilitate internalization of the suggestion, a short version was repeated during the breathing maneuvers.*

### Measurement of Suggestibility

To evaluate the dependency of results from differences in susceptibility, e.g., the ability and willingness to follow suggestions, each test person performed the Harvard Group Scale of Hypnotic Susceptibility (HGSHS) (Shor, 1962; Bongartz, 1985) in a short version in German with five items (HGSHS5:G) (Riegel et al., 2021). This is a self-assessment test, taking about 25 min. Following the common standard in suggestion or hypnosis studies the tested subjects are assigned to low, intermediate, and high suggestible, depending on the number of fulfilled items.

### Sample Size and Statistical Analysis

This study was designed as an exploratory and hypothesis generating trial with several clinically relevant endpoints. Thus, no formal a-priori sample size calculation could be performed. Nevertheless, the sample size is based on two studies in similar settings (Zech et al., 2019; Zech et al., 2020) with *n* = 50 participants or patients, respectively. We expected comparable effects throughout the endpoints and thus chose the same number of participants. As there have been relevant interindividual differences regarding absolute values of maximum respiratory strength, absolute values were set in reference to the individual baseline value. With a normal distribution of the relative parameters after suggestions, the results were reported as mean and standard deviation (SD). Differences between baseline and suggestions were analyzed using the one sample *T*-test. To analyze possible influencing variables such as age, sex, or suggestibility participants were grouped into high (HS = HGSHS5:G 4-5) and low (LS = HGSHS5:G 0-1) suggestible, male and female, as well as younger (18–39 years) and older (40–65 years). The average of all negative suggestions (negative changes in force) and all positive suggestions (positive changes in force) for the four measured parameters was compared between the groups using the Mann-Whitney *U* test, as data were not normally distributed. For better visualization, values of expiration and inspiration were grouped and differences to baseline were analysed using the one sample *T*-test. A *p* < 0.05 was considered statistically significant for all tests. Because of the exploratory character of the study, no adjustments for multiple hypotheses testing were made. All analyses were performed with IBM SPSS Statistics, Version 26.

## Results

### Baseline Characteristics

A total of 50 healthy volunteers were recruited for the study. All of them could be included in the data analyses. In a survey, 29 (58%) were women, 21 (42%) were men. The mean age was 29.1 ± 12.7 years, with a range of 18–57 years (median 23.5 years). In a survey, 40 participants were categorized as “younger” and 10 as “older.” Due to the individual physical condition of the volunteers, the baseline values had a wide range, as presented in Table 2. These values lie within the reference normal ranges, but rather at the lower limit (American Thoracic Society/European Respiratory Society, 2002; Gao et al., 2018). The baseline values were reproducible with an intra-individual variance of 5–10%, according to other publications aiming to reach 10% reproducibility (Enright et al., 1994). Additional spirometry results (i.e., FEV1, FEV) were collected and analyzed (data not shown), demonstrating unimpaired lung function for all participants. Suggestibility (HGSHS5:G) was not normally distributed, with 25 patients (50%) scoring low suggestible and seven patients (14%) high suggestible.

**TABLE 2:** Baseline values of respiratory muscle strength parameters stratified according to sex.

Parameter	Mean ± SD for male test persons (min–max) (*N* = 21)	Mean ± SD for female test persons (min–max) (*N* = 29)
MIP (cmH2O)	−77.0 ± 29.2 (−125.9–−29.1)	−50.8 ± 3.9 (−92.7–−22.3)
MEP (cmH2O)	89.1 ± 34.8 (30.3–158.4)	56.7 ± 16.8 (30.0–96.7)
PIF (L/s)	7.8 ± 2.4 (3.6–13.0)	4.4 ± 1.3 (2.4–7.0)
PEF (L/s)	9.0 ± 1.7 (5.7–12.5)	5.9 ± 1.3 (3.3–8.2)

*MIP, maximal inspiratory pressure; MEP, maximal expiratory pressure; PIF, peak inspiratory flow; PEF, peak expiratory flow; min, minimum; max, maximum.*

### Changes in Respiratory Muscle Strength After Suggestion

**FIGURE 1:**
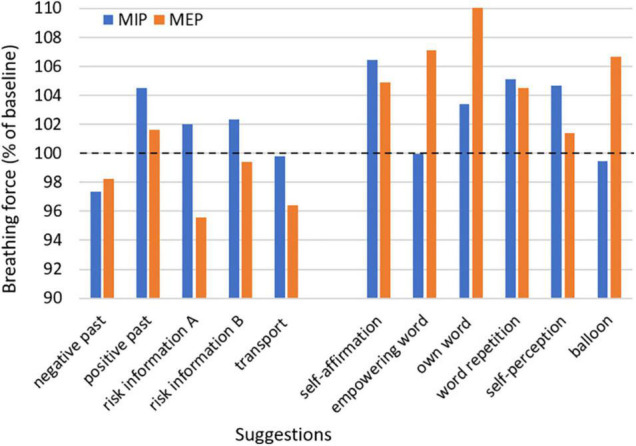
Mean of maximal inspiratory and maximal expiratory pressure after suggestions with clinical context or with a presumed strengthening effect relative to baseline. MIP, maximal inspiratory pressure; MEP, maximal expiratory pressure.

**FIGURE 2:**
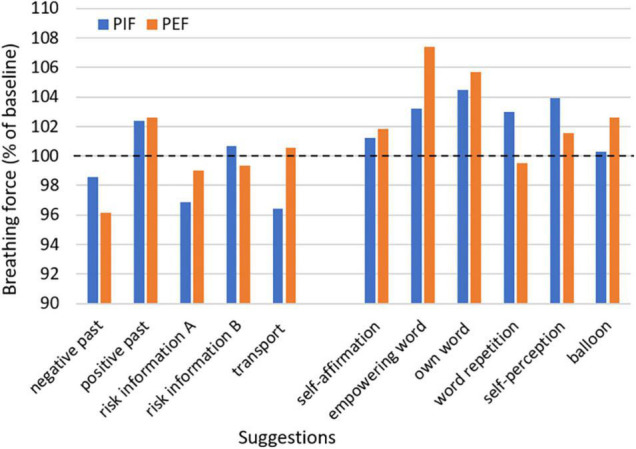
Mean of peak inspiratory and peak expiratory flow after suggestions with clinical context or with a presumed strengthening effect relative to baseline. PIF, peak inspiratory flow; PEF, peak expiratory flow.

Both suggestions with clinical context as well as suggestions intended for stimulation showed an impact on respiratory muscle strength. No single one of the measured parameters can represent muscular breathing force in its complexity; however, they can reveal trends and different specific aspects of it. The values of the breathing force after suggestions compared to baseline are given in Figures 1, 2, with pressure and flow parameters considered separately. Additionally, in Tables 3, 4 suggestions of clinical context and of stimulating intention are given separately.

**TABLE 3:** Effects of suggestions with clinical context on respiratory muscle strength parameters.

Suggestions	MIP		MEP		PIF		PEF	
	Mean ± SD	*p*	Mean ± SD	*p*	Mean ± SD	*p*	Mean ± SD	*p* *
Situation in the past								
Negative past	97.4 ± 16.3	ns	98.3 ± 16.5	ns	98.6 ± 8.0	ns	96.1 ± 13.0	**0.041**
Positive past	104.5 ± 18.2	ns	101.6 ± 13.9	ns	102.4 ± 14.8	ns	102.6 ± 10.5	ns
Risk information for informed consent								
Version A	102.0 ± 15.6	ns	95.6 ± 17.8	ns	96.9 ± 16.8	ns	99.0 ± 11.8	ns
Version B	102.3 ± 15.9	ns	99.4 ± 17.9	ns	100.6 ± 19.1	ns	99.4 ± 7.9	ns
Transport in strictly supine position	99.8 ± 13.4	ns	96.4 ± 18.4	ns	96.4 ± 14.9	ns	100.6 ± 10.0	ns

*After the determination of the baseline, suggestions were presented followed by a new measurement. Mean and SD of relative values compared to baseline (in %) after suggestions are given.*

*According to one sample T-test, results without significance are given as ns. MIP, maximal inspiratory pressure; MEP, maximal expiratory pressure; PIF, peak inspiratory flow; PEF, peak expiratory flow. Significant p-values are indicated in bold.

**TABLE 4:** Effects of intentionally strengthening suggestions on parameters of respiratory muscle strength.

Suggestions	MIP		MEP		PIF		PEF	
	Mean ± SD	*p*	Mean ± SD	*p*	Mean ± SD	*p*	Mean ± SD	*p* *
Self-affirmation	106.4 ± 20.1	**0.030**	104.9 ± 15.6	**0.035**	101.2 ± 16.3	ns	101.8 ± 11.4	ns
Empowering word								
Version A: “fireball”	100.0 ± 15.6	ns	107.1 ± 19.6	**0.013**	103.2 ± 16.9	ns	107.4 ± 9.6	**<0.001**
Version B: own power-word	103.4 ± 19.7	ns	110.3 ± 21.4	**0.001**	104.5 ± 16.9	ns	105.7 ± 10.6	**<0.001**
Version C: repetition of B	105.1 ± 20.0	ns	104.5 ± 18.9	ns	103.0 ± 13.9	ns	99.5 ± 9.9	ns
Bracing mirror image	104.7 ± 14.8	**0.029**	101.4 ± 14.6	ns	103.9 ± 15.4	ns	101.6 ± 9.3	ns
Inflating a balloon	99.4 ± 21.7	ns	106.7 ± 18.6	**0.014**	100.3 ± 14.1	ns	102.6 ± 8.8	**0.040**

*After the determination of the baseline, suggestions were presented followed by a new measurement. Mean and SD of relative values compared to baseline (in %) after suggestion are given.*

*According to one sample T-test, results without significance are given as ns. MIP, maximal inspiratory pressure; MEP, maximal expiratory pressure; PIF, peak inspiratory flow; PEF, peak expiratory flow. Significant p-values are indicated in bold.

### Effects of Suggestions With Clinical Context

Three suggestions out of everyday clinic life were tested. Two of them were presented in a presumed negative (A) and an alternative presumably neutral or positive version (B), the third was a presumably negative non-verbal suggestion. Almost every suggestion changed the pressure and flow parameters in the expected way, at least as a trend. The suggestion of a negative past (as an example of taking a patient’s medical history) decreased the values, whereas the positive past led to an increase over baseline. The strongest reaction was observed in PEF when recalling a negative situation, with a significant reduction of 3.9%. After risk information, a decrease was seen in all parameters except MIP, with the highest extend in MEP by 4.4%. On the contrary, no change from baseline was observed when positive aspects were added to the risk information (version B), and all parameters increased from version A to version B. The non-verbal suggestion of a transport in a strict supine position impaired MEP and PIF, but not MIP and PEF.

### Effects of Suggestions With Intention of Strengthening

Six suggestions were tested with the intention of increasing respiratory muscle strength (Table 4). Every suggestion resulted in a statistically significant change of at least one parameter. Expiration was affected more than inspiration, particularly noticeable for empowering words and in the case of inflating a balloon, where MIP was not affected at all. A self-affirmation resulted in a significant increase in MIP (by 6.4%) and in MEP (by 4.9%) over baseline. The effects of an empowering word were tested in three versions. The suggestion of a fixed or self-selected empowering word resulted in statistically significant higher values of the expiratory measures MEP (increase by 7.1% or 10.3%, respectively) and PEF (7.4% or 5.7%, respectively). Higher values of all parameters except PEF were achieved with the self-selected word, whereas repetition of the own word showed lower results. Participants asked to imagine a supporting or heroic mirror image of their own had an increase in parameters, predominantly in the inspiratory measures, in MIP by 4.7% with statistical significance. The imagination to inflate and fly with a balloon resulted in an improvement only in the expiratory parameters. MEP increased by 6.7% and PEF by 2.6%, both with statistical significance.

### Contributing Factors

To evaluate factors contributing to the changes induced by suggestions, the averages of all negative and of all positive suggestions were compared between high and low suggestible subjects, men and women, as well as older and younger participants (Table 5). Only sex showed an impact of statistical significance, with stronger reactions of female participants to both positive and negative suggestions. A trend was observed in the age groups, with more negative reactions of the elderly participants. Additional regression analyses between suggestion effects and suggestibility score confirmed a lack of correlation (*R*^2^ < 0.01).

**TABLE 5:** Comparison of the negative and positive average suggestion effects between groups of suggestibility, sex, and age.

Contributing factor	Average of suggestion effects	*p* *
Suggestibility (group LS vs. HS)	Positive	0.328
	Negative	0.293
Sex (male vs. female)	Positive	**<0.001**
	Negative	**0.013**
Age (group <40 vs. ≥40 years)	Positive	0.417
	Negative	0.110

*According to Mann-Whitney U test for unpaired samples. Significant p-values are indicated in bold.

## Discussion

As a main finding, verbal and non-verbal suggestions impact the clinically relevant outcome parameter breathing force. Negative suggestions, common in everyday clinical practice, have negative effects, resulting in a weakening of respiratory muscle strength. That can be avoided by alternative, neutral formulations. The application of positive suggestions improved parameters of breathing force, and therefore should be implemented in medical treatment.

### Suggestions With Clinical Context

The tested suggestion of a negative past (Situation A) is of high clinical relevance. Whenever doctors explore a medical history, they push the patient back to negative memories. This is unavoidable, but the doctor should be aware of the concomitant effects. The results of this study show that the consequence is a significant weakening of the breathing force. This confirms measurements of maximal arm muscle strength in volunteers (Zech et al., 2019) and patients (Zech et al., 2020), where the same suggestion led to a significant weakening by 10.6 and 12.9%, respectively. The reduction in muscular breathing force in this study (average change in the four parameters) was considerably lower, namely 2.5%. The clearest and statistically significant effect was observed in the reduction by 3.9% in PEF. However, also in the other parameters, there was a marked trend toward reduction. In addition, other parameters of the spirometry, namely FVC, FEV1, and MEF50, showed significant decreases by 4–5% with this suggestion (data not shown). On the contrary, talking about a positive past resulted in an increase in breathing force, by 4.5% in MIP with a significant difference to version A. These results (average change in the four parameters of 2.8%) are comparable to the increase in maximal arm muscle strength by 3.3% seen in patients (Zech et al., 2020). The extent of the strengthening effect of this suggestion must not be considered small since it is given in the medical context, which itself is rather negative. In addition, a ceiling effect can be expected, indicated by a left-sloping in the distribution of results after version “positive past,” where a further increase of maximal performance is hardly achievable. Memory of a positive past provides a solution for the weakening effect of anamnesis and a negative past. At the end of the interview about symptoms and medical history, the doctor could help the patient get out of the induced muscular impairment by asking about favorite sports before the illness or about plans for after treatment and rehabilitation (positive future).

A main source of nocebo effects is the disclosure of risk for informed consent (Miller and Colloca, 2011; Wells and Kaptchuk, 2012; Evers et al., 2018). The risk information tested for a pain catheter resulted in a slightly negative effect, highest by 4.4% in MEP. Although more prominent in the studies of arm muscle strength, nevertheless, the alternative formulation of the suggestion avoided the impairment and was neutral. Thus, the study confirms the neutralizing effect of adding a positive suggestion and expectation to the negative of risk information, for example, by addressing the benefits of the treatment offered. Other options for a positive counterweight are attempts to avoid the risks mentioned by prophylactic measures or close monitoring allowing early detection of developing adverse effects and their immediate therapy, and not least a possible own contribution of the patient to the healing process, such as careful attention or strengthening resilience (Hansen and Zech, 2019). Non-verbal suggestion during a transport in strict supine position showing only lights and ventilation ducts to patients, led to a marked decrease in PIF and MEP (by 3.6%), and no effect in PEF and MIP. In contrast to the results on arm muscle strength, the changes in breathing force observed here did not reach statistical significance. The relation between arm muscle strength and respiratory muscle strength is still unclear. That additionally raised the question, if a parameter like breathing which is so essential for life, is also sensible to suggestions. Nevertheless, the handgrip, which is also a marker for general strength, has been identified as a predictor for respiratory muscle strength (Enright et al., 1994).

In general, the study shows, as feared, nocebo effects of common medical situations also on respiration. However, together with previous studies on the same and other suggestions from everyday clinical practice, it also demonstrates that modification of the suggestions can neutralize these negative effects, and thereby communication can be improved evidence based. Because risk information given to obtain informed consent is recognized as a major source of nocebo effects, improvements are demanded (Miller and Colloca, 2011; Wells and Kaptchuk, 2012), and several proposals have been made (Evers et al., 2018; Howick, 2021). However, they have been hardly specified, tested, and validated. Some, like “framing,” have been evaluated but found to have limited efficacy (Barnes et al., 2019). The neutralization of the nocebo effect by adding positive aspects, namely the benefit of the treatment, in direct connection, as demonstrated here and in previous studies on arm muscle strength, is an example of scientific and objective proof for such an attempt.

### Stimulating Suggestions

With indications of nocebo effects, their avoidance or neutralization is not the only option and goal. There is also the option of induction of positive effects. Specifically, in this case of observing impairments of breathing force by suggestions taken from clinical context, it is about how to improve breathing. Again, this was not aimed at by suggestions targeting breathing or muscle force, or by breathing exercises, but by suggestions dealing with self-affirmation and self-strengthening, i.e., supporting healing and health in general. The question was therefore rather whether interventions directed to improve the medical situation and well-being of patients also have effects on breathing force. One of the positive suggestions tested was taken from Process- and Embodiment-focused Psychology (PEP) according to Michael Bohne (Wittfoth et al., 2020). It combines a movement of the hand and body sensation with words of self-affirmation. This intervention resulted in a marked increase in breathing force (on average by 3.8%), statistically significant in the pressure parameters MIP and MEP. Therefore, the subjective strengthening of the patients observed with this psychotherapeutic intervention was confirmed by objective measurements and parameters. This can be taken as a further example of how psychotherapeutic approaches usually traced by subjective assessments and scores can be quantified and objectified by physiological test systems (Hansen and Zech, 2019). Another intervention tested concerned the use of an empowering word. The presentation of the offered word “fireball” resulted in an increase in breathing force (on average by 4.4%), significant for the expiratory parameters MEP and PEF. The offer of an own empowering word increased the positive effect (to an average increase of 6.0%). The repetition of the chosen own word showed a reduced efficacy of this stimulating suggestion (to 3.0% on average), with a further increase only in MIP. Interestingly, a single word proved to be effective in strengthening the breath. The idea of looking into a mirror and seeing a power animal or a hero as the reflection increased breathing force, significant for MIP with an increase by 4.7%. Only one tested suggestion actually had a connection to breathing: the idea of inflating a balloon together with the metaphor of flying away with it. This intervention resulted in significant increases in the expiratory parameters PEF and MEP, with no effects on PIF and MIP. It can be taken as evidence that inspiration and expiration can be influenced separately.

### Comparison With the Literature

In a recent review on the effects of placebo and nocebo, including on physiology, the authors state that “lung function is rarely affected” (Wolters et al., 2019). All these trials have studied the effects of suggestions on dyspnoea in asthmatic patients and in some found some effects on self-reported symptoms, but no effect on measures of lung function beside bronchodilatation or bronchoconstriction (Isenberg et al., 1992). The parameters used mainly in asthmatic patients and these studies are FVC and FEV1 from spirometry and not parameters of muscular respiration performance. Others have studied the effect of suggestions in non-asthmatic healthy subjects, again with regard to bronchodilatation and bronchoconstriction and measuring respiratory resistance, not muscular components of breathing (Wigal et al., 1988). Performance of respiratory muscles was measured in a study in which pre-operative inspiratory muscle training was used in surgical patients to reduce post-operative pulmonary complications (Dronkers et al., 2008). Extensive training led to an increase in MIP by 10% (without statistical significance), while in our study the effects of verbal suggestions were immediately seen. The effect of training diminished within 6 days, while in the present study the repetition of an empowering word lost its effectiveness. Similarly, Kim and Sapienza (2005) consider MIP and MEP most appropriate to monitor training of elderly in expiratory muscle strength to improve breathing and cough to prevent atelectasis and aspiration, and call for respective studies. In a review, 24 studies were reported using expiratory muscle strength training (EMST) to increase MEP and airway protection, and to avoid post-operative pulmonary complications (Laciuga et al., 2014). However, in all these training programs and studies, verbal suggestions are not included or mentioned. All reports in the literature on suggestion, placebo and nocebo effects concerning respiration dealt with ventilation rate, bronchoconstriction, or bronchodilatation, and did not include measurements of breathing force (Wigal et al., 1988; Barber, 1996; Wolters et al., 2019). Therefore, it seems that our study is the first report of suggestion effects on the function of the breathing musculature and strength.

### Rating of Effects and Differentiation of Parameters

Most parameters of pulmonary function tests are directed at identifying restriction (impairment of lung volume) or resistance (impairment of airway by bronchoconstriction). Muscular functions in breathing can be monitored by maximal inspiratory and expiratory flows, representing the strength of abdominal and intercostal muscles, and by the respective pressures, representing the strength of the diaphragm (Chen and Kuo, 1985; Enright et al., 1994). Many studies provide evidence that respiratory muscle weakness is associated with adverse clinical outcomes, that MIP and MEP are the most appropriate parameters, and that specific training aimed at strengthening can improve outcome (Schellekens et al., 2016). This study provides evidence that verbal suggestions taken from a clinical context or aimed at supporting have an impact on the muscular components of breathing. The observed effects on breathing force were rather small and to varying extent of statistical significance, probably due to the limited number of persons tested. However, they showed a consistent trend, allowing for an evaluation and rating of the direction in which the suggestions affected persons. Even with this limitation, it should be kept in mind that the suggestions were investigated and quantified and compared using objective measures in contrast to common subjective evaluations. The weakening effect of suggestions from clinical everyday life on breathing force was less strong than on maximal arm muscle strength, which can be taken as evidence that impaired breathing as a vital function is better protected against external influences. The nature of the effects remains unclear. Some may be explained by induction of an expectation, and thus as a nocebo effect (e.g., risk information A) or a placebo effect (e.g., self-perception as a hero). Reactions to memory of a negative or positive past can be taken as a conditioning effect. Other suggestions could gain their effectiveness in other ways (Hansen and Zech, 2019). The analysis of contributing factors only showed an effect of sex, with a stronger reaction of women to both positive and negative suggestions. A similar correlation was reported when the suggestion effects were tested by maximal arm muscle strength (Zech et al., 2020). The lack of significance of suggestibility scores is not surprising and was also reported in studies of placebo/nocebo effects on the pulmonary airway, of suggestion effects on maximal arm muscle strength, and in several clinical situations (Isenberg et al., 1992; Montgomery et al., 2011; Zech et al., 2020).

**FIGURE 3:**
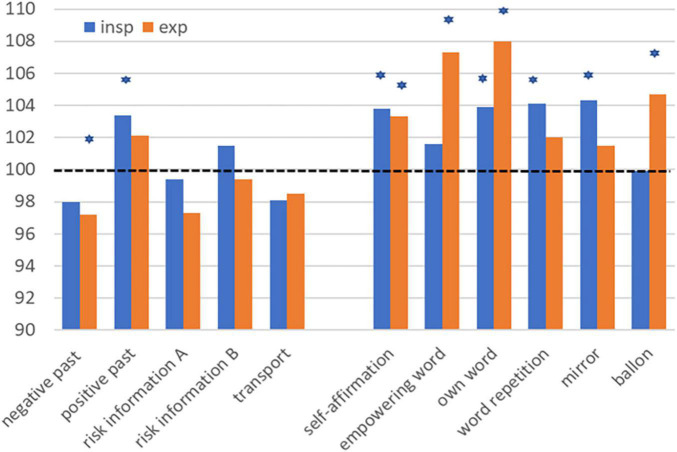
Impact of suggestions on the inspiratory (PIF, MIP) and expiratory (PEF, MEP) parameters of breathing force. MIP, maximal inspiratory pressure; MEP, maximal expiratory pressure; PIF, peak inspiratory flow; PEF, peak expiratory flow. *p < 0.05 according to one sample *T*-test.

The effects of encounters, events, and suggestions on respiration can be very different: surprises can stop our breathing, in great moments we take a deep breath, a passed danger makes us exhale with a “puh.” Consequently, it is not surprising that we observed different reactions in the various parameters of breathing force compared to the suggestions tested (Figure 3). The expiration parameters were more affected than the inspiration by memory of a negative past and the risk information for informed consent (reduction) or by an empowering word (increase). Self-strengthening by a hero mirror image had more effect on inspiration, the idea of blowing up a balloon and flying away more effect on expiration, which is expectable. Other parameters of spirometry, such as forced vital capacity (FVC) or forced one-second-capacity (FEV1) were hardly affected, especially not stimulated (data not shown). Only memory of a negative past resulted in marked and significant decreases in the latter two parameters, as an indication of a nocebo effect.

### One for All

The use of one parameter to measure, objectify, and quantify different placebo- and nocebo-effects is a unique new attempt (Hansen and Zech, 2019). Usually, the measurement is directed specifically toward the given suggestion and induced expectation. Risk information, for instance, about nausea, is followed by looking at the incidence and severity of nausea afterwards. This specificity prevents a comparison with the nocebo effect of providing risk information, i.e., on pain. What can be compared are the incidences of nausea on one side and pain on the other side. However, these are artificial categories. Nausea or pain must reach a certain level to be recognized and classified as “nausea” or “pain,” resulting in a certain number of patients with these symptoms. Is it not much more likely that in reality suggestions cause expectations and nocebo effects in all patients, though to varying degrees? This is what can be measured using an unrelated parameter like arm muscle strength or like breathing force in the present study: an uncategorized continuous range of effect. The 11 suggestions tested, except one, were not related in the sense of not being directly aimed at breathing.

In addition, the test parameters were objective measures in contrast to subjective parameters such as nausea or pain. This is even more important in the clinical context with the goal of improving communication. With a uniform parameter, the formulation and testing of alternative suggestions are supported, resulting in improvements and optimization of doctor-patient communication. Finally, using an unrelated outcome parameter turns the focus to a wider range of effects. When a suggestion induces an unspecific effect on muscle strength or breathing power, then it might be a surrogate for more general effects such as healing, immune surveillance, or resilience.

### Limitations

Studies on breathing and its muscular component breathing force are in general limited by its complexity and the lack of a unified and simple parameter. In this exploratory experimental study, only 50 healthy test persons were included. It is conceivable, that results in patients or participants with respiratory disease would have been clearer, as we have already seen testing the impact of suggestions on arm muscle strength (Zech et al., 2019; Zech et al., 2020). The limited number can explain that in spite of a rather uniform trend the results reached statistical significance only in some and varying parameters of breathing force. Furthermore, these respiration parameters show a high individual variation, which works against precision. Spirometry and measurement of respiration pressures are rather elaborate and exhausting tests, which is why the test session in this study did not exceed 1 h. For that reason, the number of items to be tested in one session is limited, making it difficult to directly compare various formulations, which would be the great advantage of using a uniform target parameter. The duration of the induced effects was not determined. Therefore, the usefulness, for example, of stimulating suggestions to support post-operative respiration remains unclear. However, the diagnostic value to identify and quantify the effects of placebo and nocebo is independent of their duration, and, in contrast, a long-lasting effect would make it impossible to test and compare several suggestions in one test session.

### Conclusion

The evaluation of placebo and nocebo effects is often limited because the measurement parameter is chosen according to the specific suggestion (e.g., pain is measured following a procedure suggested to be painful), and other effects are not monitored, or subjective measures are used like pain score, feeling of nausea, or itch. Besides, often graduated parameters are used that categorize the effect (e.g., the number of patients with a certain symptom is evaluated in the verum and the placebo group), and continuous effects, i.e., smaller effects in the other patients are ignored.

Using physiological parameters such as maximal arm muscle strength or in this study spirometry and breathing pressures allows objective measurement of a continuous variable in all tested persons, evaluation and comparison of different suggestions, and extending the view from specific to more general effects. The most important finding is, that the breathing force is a clinically relevant parameter which can be influenced in both directions with suggestions. There is the need to avoid or neutralize impairments and indications to support and enhance this function. This study can sensitize anyone working in medical fields, that the way we communicate has impact on the patient and might influence the outcome. Accordingly, positive suggestions should be used, and negative ones avoided. Further research should study the impact of suggestions on other physiological parameter and evaluate our findings in patients.

## Data Availability Statement

The original contributions presented in the study are included in the article/Supplementary Material, further inquiries can be directed to the corresponding author.

## Ethics Statement

The studies involving human participants were reviewed and approved by EC University of Regensburg. The participants provided their written informed consent to participate in this study.

## Author Contributions

NZ: study design, application for ethic committee approval, literature search, participant recruitment, data collection and analysis, and preparation of the manuscript. LS: participant recruitment, data collection and analysis, and correction of the manuscript. MS: study design and application for ethic committee approval. MP: literature search and preparation of manuscript. EH: study plan and design, supervision, literature search, data analysis, preparation of figures, tables and manuscript, and correction of manuscript. All authors contributed to the article and approved the submitted version.

## Conflict of Interest

The authors declare that the research was conducted in the absence of any commercial or financial relationships that could be construed as a potential conflict of interest.

## Publisher’s Note

All claims expressed in this article are solely those of the authors and do not necessarily represent those of their affiliated organizations, or those of the publisher, the editors and the reviewers. Any product that may be evaluated in this article, or claim that may be made by its manufacturer, is not guaranteed or endorsed by the publisher.
