# Supplementary material for: Nocebo Effects of Clinical Communication and Placebo Effects of Positive Suggestions on Respiratory Muscle Strength
Source: Front Psychol. 2022 Mar 11;13:825839. doi: 10.3389/fpsyg.2022.825839 (PMC8962828; doi:10.3389/fpsyg.2022.825839)
Supplement: Supplementary file 1 [file Table_1.DOCX]

STROBE Statement—checklist of items that should be included in reports of observational studies

# Item

**No Recommendation**

| **Title and abstract** | 1 | (*a*) Indicate the study’s design with a commonly used term in the title or the abstract-page 1 |
| --- | --- | --- |
|  |  | (*b*) Provide in the abstract an informative and balanced summary of what was done  and what was found- page 2,3 |
| **Introduction** |  |  |
| Background/rationale | 2 | Explain the scientific background and rationale for the investigation being reported-page 4, 5 |
| Objectives | 3 | State specific objectives, including any prespecified hypotheses-page 5 |
| **Methods** |  |  |
| Study design | 4 | Present key elements of study design early in the paper-page 6 |
| Setting | 5 | Describe the setting, locations, and relevant dates, including periods of recruitment,  exposure, follow-up, and data collection-page 6,7 |
| Participants | 6 | (*a*) *Cohort study*—Give the eligibility criteria, and the sources and methods of selection of participants. Describe methods of follow-up  *Case-control study*—Give the eligibility criteria, and the sources and methods of case ascertainment and control selection. Give the rationale for the choice of cases and controls  *Cross-sectional study*—Give the eligibility criteria, and the sources and methods of  selection of participants-page 6 |
|  |  | (*b*) *Cohort study*—For matched studies, give matching criteria and number of exposed and unexposed- na  *Case-control study*—For matched studies, give matching criteria and the number of  controls per case- na |
| Variables | 7 | Clearly define all outcomes, exposures, predictors, potential confounders, and effect  modifiers. Give diagnostic criteria, if applicable-page 9,10 |
| Data sources/ measurement | 8* | For each variable of interest, give sources of data and details of methods of assessment (measurement). Describe comparability of assessment methods if there  is more than one group-page 6,7 |
| Bias | 9 | Describe any efforts to address potential sources of bias-na |
| Study size | 10 | Explain how the study size was arrived at-page 10 |
| Quantitative variables | 11 | Explain how quantitative variables were handled in the analyses. If applicable,  describe which groupings were chosen and why-page 10 |
| Statistical methods | 12 | (*a*) Describe all statistical methods, including those used to control for confounding-page 10 |
|  |  | (*b*) Describe any methods used to examine subgroups and interactions-page 10 |
|  |  | (*c*) Explain how missing data were addressed- na |
|  |  | (*d*) *Cohort study*—If applicable, explain how loss to follow-up was addressed  *Case-control study*—If applicable, explain how matching of cases and controls was addressed  *Cross-sectional study*—If applicable, describe analytical methods taking account of  sampling strategy na |
|  |  | (*e*) Describe any sensitivity analyses- na |
| Continued on next page |  |  |

**Results**

Participants 13*

1. Report numbers of individuals at each stage of study—eg numbers potentially eligible, examined for eligibility, confirmed eligible, included in the study, completing follow-up, and analysed-page 11
2. Give reasons for non-participation at each stage na
3. Consider use of a flow diagram na

Descriptive data

14*

1. Give characteristics of study participants (eg demographic, clinical, social) and information on exposures and potential confounders-page 11
2. Indicate number of participants with missing data for each variable of interest-na
3. *Cohort study*—Summarise follow-up time (eg, average and total amount)

Outcome data 15*

Main results 16

*Cohort study*—Report numbers of outcome events or summary measures over time

*Case-control study—*Report numbers in each exposure category, or summary measures of exposure

*Cross-sectional study—*Report numbers of outcome events or summary measures-Table 3 and 4

1. Give unadjusted estimates and, if applicable, confounder-adjusted estimates and their precision (eg, 95% confidence interval). Make clear which confounders were adjusted for and why they were included-na
2. Report category boundaries when continuous variables were categorized-na
3. If relevant, consider translating estimates of relative risk into absolute risk for a meaningful time period-na

Other analyses 17 Report other analyses done—eg analyses of subgroups and interactions, and sensitivity analyses-Table 5

# Discussion

Key results 18 Summarise key results with reference to study objectives-page 17

Limitations 19 Discuss limitations of the study, taking into account sources of potential bias or imprecision.

Discuss both direction and magnitude of any potential bias-page 24, 25

Interpretation 20 Give a cautious overall interpretation of results considering objectives, limitations, multiplicity of analyses, results from similar studies, and other relevant evidence-page 20,21

Generalisability 21 Discuss the generalisability (external validity) of the study results-page 23,24

# Other information

Funding 22 Give the source of funding and the role of the funders for the present study and, if applicable, for the original study on which the present article is based-no funding

*Give information separately for cases and controls in case-control studies and, if applicable, for exposed and unexposed groups in cohort and cross-sectional studies.

**Note:** An Explanation and Elaboration article discusses each checklist item and gives methodological background and published examples of transparent reporting. The STROBE checklist is best used in conjunction with this article (freely available on the Web sites of PLoS Medicine a[t http://www.plosmedicine.org/,](http://www.plosmedicine.org/) Annals of Internal Medicine at [http://www.annals.org/,](http://www.annals.org/) and Epidemiology at [http://www.epidem.com/).](http://www.epidem.com/)) Information on the STROBE Initiative is available at [www.strobe-statement.org.](http://www.strobe-statement.org/)
